# Myeloproliferative Disease: An Unusual Cause of Raynaud's Phenomenon and Digital Ischaemia

**DOI:** 10.1155/2016/9675171

**Published:** 2016-11-08

**Authors:** Celia Beynon, Gwenan Huws, Tom Lawson

**Affiliations:** ^1^Department of Rheumatology, Princess of Wales Hospital, Coity Road, Bridgend CF31 1RQ, UK; ^2^Department of Rheumatology, Royal Gwent Hospital, Cardiff Road, Newport NP20 2UB, UK

## Abstract

We describe a 59-year-old female who presented with ischaemic digits, preceded by a 6-month history of Raynaud's phenomenon affecting her fingers and toes. There were no clinical or laboratory features of primary vasculitis or connective tissue disease, Doppler imaging was normal, and bloods were unremarkable aside from a platelet count of 786 × 109/L (150–400) and white cells of 16 × 109/L (4–11). In view of the thrombocytosis a JAK2 mutation assay was requested which confirmed a JAK2 V617F mutation, suggesting essential thrombocytosis (ET) as the cause. She received treatment with hydroxycarbamide which normalised her platelet count and led to a complete resolution of her Raynaud's symptoms. Raynaud's phenomenon is a rare manifestation of ET. Myeloproliferative disorders such as ET should be considered in the differential diagnosis of Raynaud's phenomenon and vasculitis.

## 1. Introduction

Raynaud's phenomenon is a common symptom encountered by general practitioners and hospital doctors. It is commonly a primary disorder but can be secondary to other conditions. Whilst the underlying cause in this case was unusual it emphasizes the importance of considering secondary causes which may not have an autoimmune aetiology.

## 2. Case Report

A 59-year-old was referred to the rheumatology clinic for investigation of possible vasculitis. She had a two-week history of pain and discolouration of her left second and third toes preceded by six months of Raynaud's phenomenon affecting her fingers and toes. She experienced several attacks of Raynaud's each day often triggered by cold. She was otherwise well with no symptoms to suggest an underlying connective tissue disease and no past medical history of note. She had recently stopped smoking, having previously smoked 20 cigarettes daily for more than 20 years.

**Figure 1:**
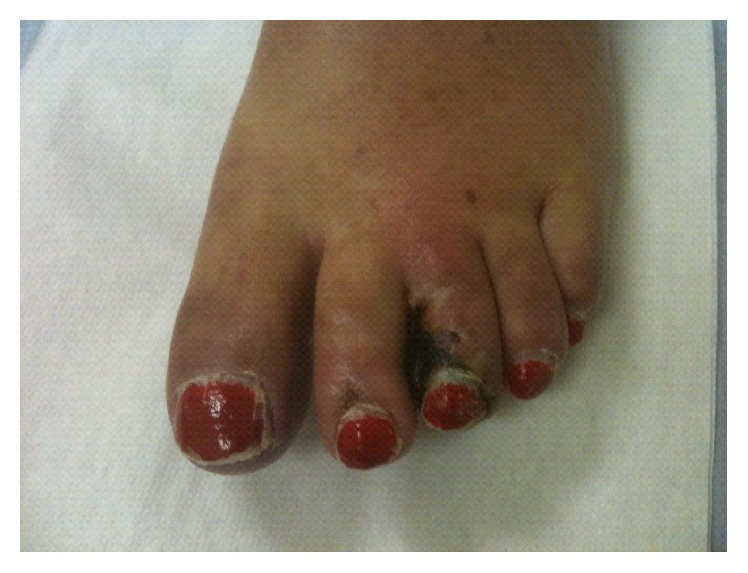
Left foot of a 59-year-old patient presenting to rheumatology clinic.

On examination she was apyrexial with ischaemic lesions on 2nd and 3rd toes (Figure 1). Peripheral pulses were normal and systemic examination was unremarkable. Urinalysis was negative.

Investigations revealed haemoglobin of 146 g/L (115–165), platelet count of 786 × 10^9^/L (150–400), and white cells of 16 × 10^9^/L (4–11). White cell differential showed a lymphocyte count of 3.8 × 10^9^/L (1.5–4.5), neutrophil count of 10.1 × 10^9^/L (1.7–7.5), monocytes of 1.2 × 10^9^/L (0.2–0.8), eosinophils of 0.8 × 10^9^/L (0–0.4), and basophils of 0.2 × 10^9^/L (0–0.1). Liver, renal, and thyroid functions were normal as were CRP, immunoglobulins, complement levels, and Doppler imaging of the lower limbs. ANA, ANCA, and rheumatoid factors were negative.

In view of the thrombocytosis, a JAK2 mutation assay was performed. This confirmed a JAK2 V617F mutation suggesting essential thrombocytosis (ET) as the cause.

Initially treatment was directed at improving peripheral blood flow and pain control. This included oral nifedipine, aspirin, and intravenous iloprost. Opiates were required for pain relief. Despite these measures the toe failed to improve and the Raynaud's symptoms continued.

Following the diagnosis of ET treatment with hydroxycarbamide was commenced. Treatment with hydroxycarbamide normalised the platelet count within three months and led to complete resolution of the Raynaud's symptoms and healing of the ischaemic lesions.

## 3. Discussion

ET is an uncommon myeloproliferative disease with an estimated incidence of 0.4–2.5 per 100,000 per year [1]. A typical patient is female and diagnosed in the 5th or 6th decade of life [2]. The JAK2 V617F mutation is identified in 50–60% of cases of ET [3].

Patients with ET may present with haemorrhagic complications, due to platelet dysfunction, or thrombotic disorders including stroke, coronary artery ischaemia, deep vein thrombosis, and digital ischaemia. Thrombotic events occur more frequently in patients with ET who are positive for the JAK2 V617F mutation [3]. The platelet count is often elevated above 1000 × 10^9^/L although studies suggest a median level of around 800 × 10^9^/L at presentation [4]. Lower levels are not unusual and may be attributed to inflammation or tissue necrosis.

Our patient presented with the triphasic white, blue, and red colour change of Raynaud's and features of digital ischaemia. It is more common for patients to present with erythromelalgia (EM) [5, 6]. EM is characterised by redness and painful burning of the extremities with symptoms aggravated by heat or exercise and relieved by cold. In a retrospective study of 268 ET patients, 15 had features of EM, but Raynaud's phenomenon was only observed in 1 patient [5].

The underlying pathology of EM is thought to be occlusion of the microcirculation by platelets, which is also likely to be a factor in ET associated Raynaud's.

## 4. Conclusion

Myeloproliferative disorders such as ET should be considered in the differential diagnosis of vasculitis and secondary Raynaud's phenomenon. Investigation of suspected secondary Raynaud's phenomenon should include full blood count, renal function, urinalysis, immunoglobulins, ANA, and ANCA. Treatment of the underlying disorder can lead to resolution of symptoms and signs
